# Prevalence and Clinical Significance of Occult Fractures in the Extremities in Children

**DOI:** 10.3389/fped.2020.00393

**Published:** 2020-08-04

**Authors:** Qichao Ma, Qin Jiao, Shiqi Wang, Liangchao Dong, Yicheng Wang, Mengjie Chen, Sun Wang, Hao Ying, Lihua Zhao

**Affiliations:** Department of Orthopedics, Shanghai Children's Hospital, Shanghai Jiao Tong University, Shanghai, China

**Keywords:** occult fracture, prevalence, distribution, children, orthopedic trauma

## Abstract

**Objective:** Diagnosis of occult fractures by initial plain radiographs remains challenging in children in the emergency room. This study was to assess the prevalence and distribution of occult fracture in children with acute extremities injuries (AEI) and clinical suspicion of fracture.

**Methods:** We conducted a retrospective study to review the medical records of all pediatric patients with AEI in the orthopedic emergency room from January 1, 2017, to December 31, 2019. For patients with concerning history and physical examination but negative initial radiographs, we conducted the following three diagnostic strategies according to the choic of children's parents: immediate MRI scanning, [2] immediate CT scanning, or [3] empiric cast immobilization with orthopedic follow-up radiographs at 2 weeks post-injury (late radiographs). Prevalence and distribution of occult fracture were recorded.

**Results:** A total of 43,560 pediatric patients meet the inclusion criteria. A total of 4,916 fractures of the extremities were confirmed by initial plain radiographs, and 550 occult fractures were confirmed by immediate MRI, immediate CT, or late radiographs. The prevalence of occult fracture in the extremities was 10.1% (550/5,466). Supracondylar fractures were the most prevalent (2,325/5,466, 42.5%) but had the lowest rate of occult fractures (117/2,325, 5.0%). The highest rate of occult fracture was distal epiphyseal fracture of the tibia and fibula (49/145, 33.8%), but these had a relatively lower prevalence of fractures (145/5,466, 2.65%).

**Conclusions:** We should be aware of the relative high prevalence of occult fractures in the extremities in children, especially when the injured site is in the high incidence area of occult fracture such as ankle.

## Introduction

Acute extremity injuries (AEI) are very common in children. They may constitute up to 90% of orthopedic emergency department (ED) visits and comprise approximately 85% of all injuries to the musculoskeletal system in children (1, 2). The diagnosis of fractures involves history and physical examination as well as radiographs. Pediatric orthopedic surgeons traditionally use plain radiographs to exclude fractures when there is suspicion of a fracture in children with AEI, as whether there is a fracture is the primary concern of parents and clinicians. However, interpretation of plain radiographic images of childhood AEI is challenging in comparison to adults, and plain radiographs may fail to reveal a fracture because of a child's developmental and anatomical characteristics, such as the presence of a secondary ossification center, additional areas of ossification, and an open physeal plate (3). As a result, minor fractures or fractures that are not easily detected (occult fracture) may be missed.

An occult fracture represents a type of fracture that cannot be detected by radiography or which shows subtle abnormalities that were missed on the initial radiograph, even if the fracture is visualized retrospectively or confirmed by other imaging methods (4, 5). The prevalence of occult fractures in children has been reported to be 2–25% of reviewed cases (6). Under-treatment of children with fracture or over-treatment of children without fracture may occur, and lead to medical (7), psychosocial (8), financial (2), and legal consequences (9) to patients and clinicians.

Although there is no accepted gold standard of diagnosis for occult fracture, pediatric orthopedic surgeons traditionally rely on the use of late follow-up radiographs (10), magnetic resonance imaging (MRI), or computed tomography (CT) as the reference standard to obtain an accurate diagnosis, especially on MRI or CT (11). Berger et al. reported that MRI provides valuable information about soft tissue abnormalities, particularly ligamentous lesions, and posttraumatic bone marrow changes, but sometimes a fracture line can be difficult to analyze (4). MRI has 99% sensitivity in detection of occult fractures because of its high resolution, and it is extremely helpful for the detection of occult fractures and soft tissue abnormalities, especially ligamentous injuries in cases of AEI (1–3, 12). However, high costs, limited availability, and long duration of the MRI examination limit wide application of MRI for AEI, especially in children. Compared with MRI, CT is more readily available and convenient for pediatric patients and produces a more accurate description of subtle fracture lines, depressed or distracted articular surfaces, and better evaluation of bone loss (11, 13, 14). However, CT cannot provide information about soft tissue changes or bone marrow edema and involves high doses of radiation (6, 13). Studies have demonstrated that ultrasound (US), when performed by trained and experienced operators, is an effective tool for detecting occult or missed fractures in children as it is a valuable method for distinguishing joint effusion, soft tissue, and bone surface (15). In addition, it is readily available, cost-effective, and radiation-free. US has been reported to show good diagnostic accuracy in diagnosis of occult ankle fractures, occult radial head and neck fractures, and occult scaphoid fractures when initial radiographs showed only intraarticular effusion (15, 16).

To date, no study has reported the prevalence of occult fractures in children with radiograph-negative AEI and clinical suspicion of fracture. We therefore conducted a retrospective study to assess the prevalence and distribution of occult fractures in children with AEI in our orthopedic ED room between 2017 and 2019.

## Patients and Methods

This was a retrospective study which was approved by the Institutional Review Board of our hospital. We reviewed the cases of pediatric patients who visited our orthopedic ED due to AEI from January 1, 2017, to December 31, 2019. The inclusion criteria included children aged under 14 years old, with acute extremity injuries, who initially visited our orthopedic ED. Exclusion criteria included fractures confirmed in other hospitals, fractures at multiple sites, or injuries to the skull, spine, or pelvis. Patients first had their full medical history taken and then were submitted to clinical examination and standard radiographs (anteroposterior and lateral view). Patients then underwent immediate MRI, immediate CT, or late follow-up radiographs 2 weeks post-injury according to the willingness of the children's parents when there was clinical suspicion of fracture but no confirmation of fracture on plain radiographs.

If the children's parents chose late follow-up radiographs 2 weeks post-injury, empiric cast immobilization was performed for the patients. For displaced or surgically reduced fractures, control standard radiographs were taken weekly for 1 month to analyze fracture healing and bone alignment; for stable fractures, control standard radiographs were taken every 2 weeks for 1 month to observe the above conditions; for greenstick fractures, control standard radiographs were conducted 1 month after injury.

At least two pediatric radiologists retrospectively reviewed all original plain radiographs and reports. We calculated the total number of each type of fracture (including occult fractures) in the extremities. Occult fracture was defined as an injured child with negative plain radiographs (radiographically undetectable or shows subtle abnormalities) but clinical suspicion of fracture, confirmed as fracture by immediate MRI, immediate CT, or late radiographs 2 weeks post-injury (4).

To estimate the correlation between the prevalence of fractures of the extremities and the prevalence of occult fractures, we used Pearson's correlation coefficient to analyze the statistical data by SPSS version 20 (IBM, Armonk, NY, USA). A two-tailed value of *P* < 0.065 indicated significance.

## Results

**Figure 1:**
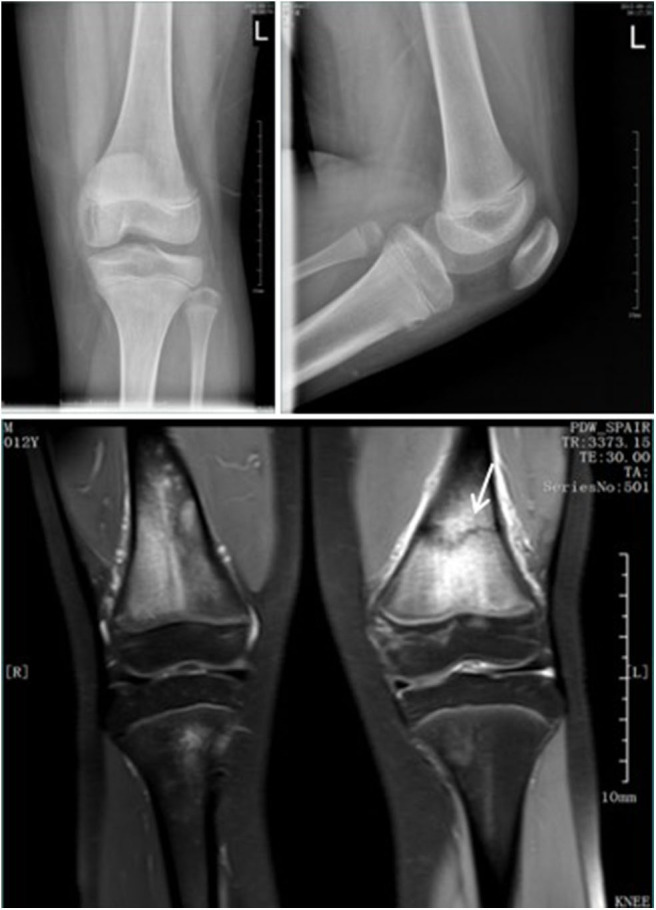
Standard radiographs in AP and LL projections did not show a fracture. MRI demontrated a fracture in the distal femur (arrow).

**Figure 2:**
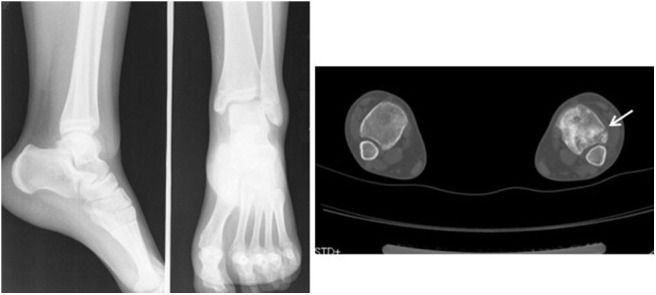
Standard radiographs of the ankle in AP and LL projections did not reveal a fracture. CT showed a small fracture in the distal fibula (arrow).

**Figure 3:**
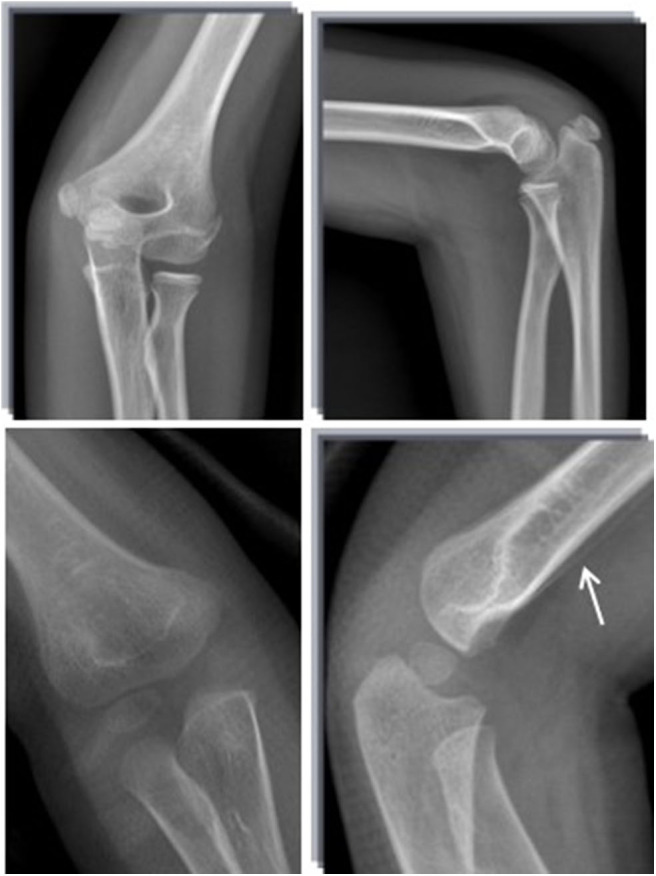
Standard radiograph in AP and LL projections. Fractures were not visible at the first visit. After 3 weeks, a healing line (arrow) could be seen clearly.

**Figure 4:**
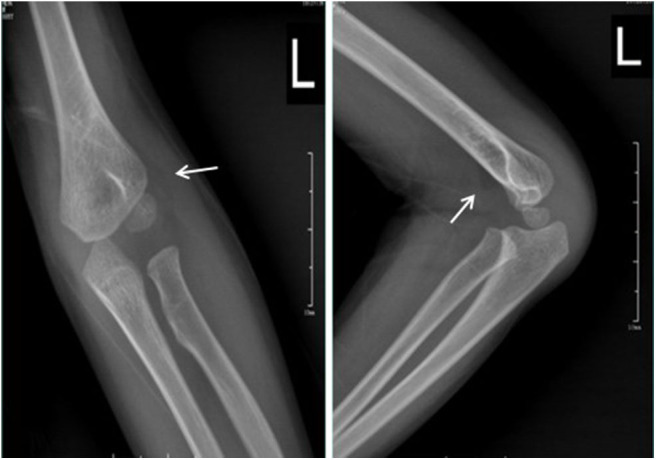
Standard radiographs in AP and LL projections showing anterior and posterior fat pads (arrow).

A total of 43,560 pediatric patients with AEI were enrolled, of whom 5,466 patients were diagnosed with fracture, 4,916 of which were confirmed by initial plain radiographs, and 550 were occult fractures confirmed by immediate MRI, immediate CT, or late radiographs 2 weeks post-injury. The mean age of the occult fracture patients was 7.9 years (range 1.6–14 years). The distribution of fractures diagnosed by initial plain radiographs and the distribution and rate of occult fractures in the extremities is summarized in Table 1. The prevalence of occult fractures of the extremities was 10.1% (550/5,466). The history and physical examination findings of all 550 patients led the orthopedic surgeons to suspect a possible fracture although there were no visible signs of fracture on the initial anteroposterior and lateral radiographs. Immediate MRI (*n* = 193) (Figure 1), immediate CT (*n* = 199) (Figure 2), or late radiographs (*n* = 158) (Figure 3) finally confirmed these occult fractures. The physical examination of the 550 patients revealed abnormal soft tissue swelling, persistent pressure pain at the trauma site, and limited active and passive motion because of pain. For patients with distal humerus injury, their anteroposterior and lateral radiographic views showed displacement of the fat pads (fat pad sign) due to joint effusion, which is an indirect sign of fracture (Figure 4).

**Table 1:** The distribution of fractures diagnosed by initial plain radiographs and the distribution and rate of occult fractures in the extremities detected in orthopedic AEI children.

	**Fracture confirmed by initial plain radiographs**	**Occult fracture**	**Total fracture**	**Rate of occult fracture (%)**
Supracondylar fracture	2,208	117	2,325	5.0
Lateral condyle fracture	808	101	909	11.1
Tibial fracture	450	24	474	5.1
Olecranon fracture	275	50	325	15.4
Fracture of foot and hand	461	28	489	5.7
Distal epiphyseal fractures of tibia and fibula	96	49	145	33.8
Distal epiphyseal fractures of radius and ulna	243	48	291	16.5
Radial neck fractures	147	59	206	28.6
Monteggia fractures	121	39	160	24.4
Humeral medial epicondyle fractures	50	11	61	18.0
Fractures of the epiphyseal end of distal femur	36	17	53	32.1
Intraarticular knee fractures	21	7	28	25.0
Total	4,916	550	5,466	10.1

The rate of occult fractures in major bones of the extremities is summarized in Table 2. Seventy-three occult tibia and fibula fractures were identified (73/550, 13.3%), 67.1% (49/73) of which were distal tibia and fibula epiphyseal fractures and five of them were treated surgically (Salter-Harris II fracture: three cases; Salter-Harris III fracture: two cases; the age of all five operated patients was >8 years old), and 32.9% (24/73) were occult tibial fractures. No occult wrist fractures were detected in this study.

**Table 2:** Rate of occult fractures in major bones of the extremities detected in orthopedic AEI children.

**Occult fracture**	**Number**	**Rate (%)**
Humerus	229	41.64
Radius and ulna	196	35.64
Tibia and fibula	73	13.27
Foot and hand	28	5.09
Femur	17	3.09
Knee	7	1.27
Total	550	100

**Figure 5:**
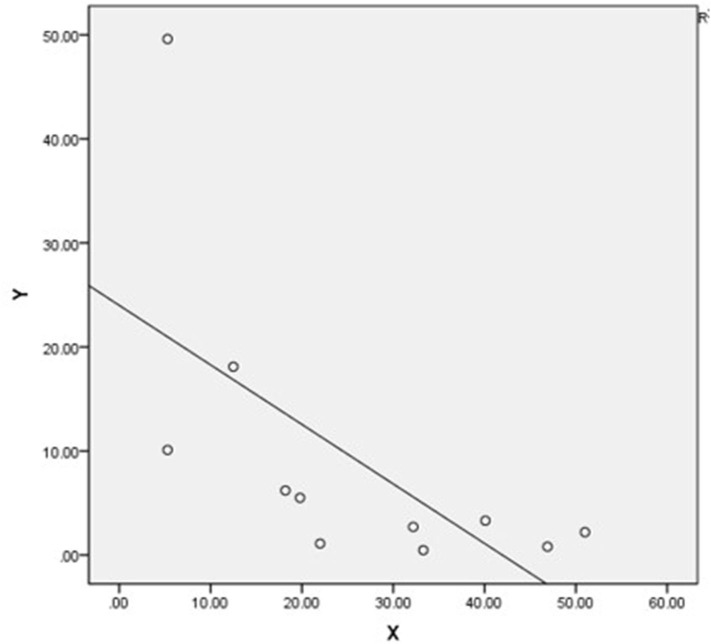
Scatter plot of the prevalence of fractures of the extremities (Y) and prevalence of missed occult fractures (X) shows a significantly negative linear relationship (*P* = 0.04, r = −0.58).

Supracondylar fractures were the most prevalent (2, 325/5, 466, 42.5%) but had the lowest rate of occult fractures (117/2, 325, 5.0%). The highest rate of occult fracture was distal epiphyseal fracture of the tibia and fibula (49/145, 33.8%), but these had a relatively lower prevalence of fractures (145/5, 466, 2.65%) (Table 1). The Pearson's correlation analysis indicated a significantly negative linear relationship between the prevalence of fractures and the rate of occult fractures of the extremities (*P* = 0.04, *r* = −0.58) (Figure 5).

## Discussion

The term “occult fracture” is defined broadly as a radiographically undetectable or subtle abnormality that has been missed on initial radiographs (4). The abnormalities which can indicate possible fractures include the fat pad sign because of joint effusion, immobility, stable pressure pain point, and abnormal swelling (17, 18). We have not found any previous study that has attempted to determine the prevalence of occult fractures and their clinical significance in children with radiograph-negative AEI and clinical suspicion of fracture.

Consequently, a total of 43,560 patients were analyzed; 550 of these patients had occult fracture and the prevalence of occult fractures was 10.1% (550/5, 466) among AEI children. Najaf-Zadeh et al. reviewed nine studies with 187 patients, and their meta-analysis of the prevalence of occult fractures in children with radiograph-negative acute ankle injuries was 24% (6). Boutis et al. conducted a multicenter study in Boston and Toronto and found a 1% (6/607) prevalence of distal tibial and fibular occult fractures (5), which is much higher than our finding (49/43, 560; 0.1%). The incidence of occult fractures reported in the literature differs greatly from that found in our study, which may be related to our large sample size. Moreover, we looked at patients with AEI, not patients with injury to only one part of the body such as the ankle. In our study, Pearson's correlation analysis indicated a significantly negative linear relationship between the prevalence of fractures and the rate of occult fractures of the extremities, which suggested that pediatric orthopedic surgeons should be highly vigilant in the clinic, as occult fractures are more likely to be missed in areas with a lower incidence of fractures, such as the ankle (Figure 5). This study identified the common sites where occult fractures occur. For these sites, if a fracture is suspected, the treatment plan of reexamination after 2 weeks of immobilization and fixation may be preferred to avoid the need for further CT or MRI. We found that occult fractures were found less frequently at sites where fractures occur frequently, the reason for which may be related to children's developmental and anatomical characteristics, such as secondary ossification centers, additional areas of ossification, and open physeal plates (3). It is challenging to spot a fracture or interpret radiographic imaging near the growth plate in children as ossification of the epiphysis has not yet taken place or is incomplete. The anatomy of some specific locations, such as the ankle, was complicated, and we were unable to find fractures based solely on plain radiographs at these sites. In our study, five occult distal tibia and fibula epiphyseal fractures were operated on, and the patient's age was >8 years in all cases; we therefore recommend using immediate CT or immediate MRI for the detection of occult fractures in patients who have suffered a serious ankle sprain, especially for those aged >8 years with a swollen ankle (19). Distal tibia and fibula epiphyseal Salter-Harris II, III, or IV fractures, which are sometimes not visible on plain radiographs, require timely surgical internal fixation to prevent the occurrence of long-term complications such as early closure of the epiphyses and formation of bone bridges. We also found 39 occult Monteggia fractures (Table 1), 18 of which were missed because of incorrect X-ray positioning, while 11 were really occult Monteggia fractures (Bado Type II and Bado Type III), and 10 were Bado type I equivalents; this result reminds us of the importance of radiographic positioning and the different types of Monteggia fractures.

In cases of joint effusion, the anterior fat pad is elevated anteriorly, and this is known as a “positive anterior fat pad sign.” Studies have shown that a visible posterior fat pad sign and history of acute trauma are likely to indicate intra-articular fractures with a predictive value of 75%. We also observed this fat pad sign on radiographs, especially in cases of elbow trauma (Figure 4). If the first plain radiograph does not show a fracture, indirect signs of fracture should be looked for (17, 18). However, a visible anterior fat pad might be normal if it is close to the bone (17). US is effective, radiation-free, and convenient in detecting occult fractures in the orthopedic ED (15), especially for occult radial head and neck fractures and occult scaphoid fractures when initial radiograms show only intra-articular effusion (15, 16). We observed a good diagnostic accuracy of late radiographs, MRI, and CT for diagnosing occult AEI fractures, but we did not use US in our study, and this is a limitation of our study; we will evaluate the use of US for diagnosing occult AEI fractures in the future.

It is very important for AEI patients to obtain an accurate diagnosis. The discrepancy between clinical and radiographic findings could lead to under-treatment or over-treatment. Kan et al. retrospectively reviewed the cases of 204 children with suspected extremity fractures and negative initial radiographs; 29 (29/204, 14.2%) of these children had a fracture identified on follow-up radiographs, while 102 of them had no fracture and were over-treated, and 68 of the children with fracture were under-treated (2). In our study, 550 of 43,560 (1.3%) AEI patients were found to have occult fractures. Accordingly, approximately 10% of these children were at risk of under-treatment if assessment was based solely on initial plain radiographs, and 90% were at risk of over-treatment if based solely on clinical grounds. The instances of misdiagnosis and missed diagnoses not only lead to under-treatment or over-treatment, which could affect patients' quality of life and limb function, but also lead to disputes between hospitals and patients. The number of disputes taking place in the EDs of full-service tertiary hospitals is three times that seen in secondary hospitals. The most common causes of violence were dissatisfaction with the treatment or the diagnosis (51%) (20), and most of these disputes were in surgery- or pediatric-related departments (21). Thus, it is necessary to obtain an accurate diagnosis for AEI patients.

In order to clarify the diagnosis and avoid or reduce the risk of under-treatment or over-treatment, it is necessary to perform further measures such as late radiographs (22), MRI, or CT when clinical findings are suggestive of fracture but plain radiographs are negative. Radiation exposure was a very important concern for our patients or their parents in deciding which methods to choose (late radiographs, immediate MRI, or immediate CT) to clarify the diagnosis. In our study, we used late radiographs, immediate MRI, or immediate CT as the reference standard and confirmed their affectivities in detecting occult fractures in children, and we highly recommended those adolescent patients with swollen sprained ankle to accept further MRI or CT examination. We explained to parents the pros and cons of each method, but the ultimate choice of method was up to the parents themselves. The cost of MRI is lower in China (around 70 dollars per part of body), which is generally acceptable to families. Coupled with the high radiation of CT, in cases where it is difficult to rule out occult fracture, while following the parents' preferences, MRI is recommended to avoid radiation exposure and obtain a definite diagnosis. We did not compare the results of standard radiographs with MRI or CT results and thus could not reach a conclusion regarding the best choice between these methods as an orthopedic surgeon, which is a question we will address in a future study.

## Conclusion

In conclusion, radiographically-occult and subtle fractures of the extremities are often a challenging diagnostic problem in daily clinical practice. Our findings provide evidence that in children with AEI and clinical suspicion of fracture, a substantial proportion of fractures could be missed initially because radiographic evidence of such fractures may not appear until weeks after the initial injury. When the fracture line is not clearly visible on standard radiographs and there are indirect signs of fracture, additional imaging methods are required. Late radiographs, immediate MRI, and immediate CT appear to be promising imaging modalities to detect occult fractures in children with AEI.

## Data Availability Statement

The raw data supporting the conclusions of this article will be made available by the authors, without undue reservation, to any qualified researcher.

## Ethics Statement

The studies involving human participants were reviewed and approved by Shanghai Children's Hospital of Ethics Committee.

## Author Contributions

QJ, QM, and LZ conceived and designed the study. YW, MC, SW, and LD collected the data. QM, QJ, LZ, and HY analyzed and interpreted the data. LZ and QM wrote and revised the paper. All authors read and approved the final draft, approved the final manuscript as submitted, and agree to be accountable for all aspects of the work.

## Conflict of Interest

The authors declare that the research was conducted in the absence of any commercial or financial relationships that could be construed as a potential conflict of interest.
